# Risk Factors for Treatment Default among Re-Treatment Tuberculosis Patients in India, 2006

**DOI:** 10.1371/journal.pone.0008873

**Published:** 2010-01-25

**Authors:** Ugra Mohan Jha, Srinath Satyanarayana, Puneet K. Dewan, Sarabjit Chadha, Fraser Wares, Suvanand Sahu, Devesh Gupta, L. S. Chauhan

**Affiliations:** 1 Central Tuberculosis Division, Directorate General of Health Services, Ministry of Health and Family Welfare, Government of India, New Delhi, India; 2 Centre for Operational Research, International Union Against Tuberculosis and Lung Diseases (The Union), New Delhi, India; 3 Office of the WHO Representative to India, New Delhi, India; McGill University, Canada

## Abstract

**Setting:**

Under India's Revised National Tuberculosis Control Programme (RNTCP), >15% of previously-treated patients in the reported 2006 patient cohort defaulted from anti-tuberculosis treatment.

**Objective:**

To assess the timing, characteristics, and risk factors for default amongst re-treatment TB patients.

**Methodology:**

For this case-control study, in 90 randomly-selected programme units treatment records were abstracted from all 2006 defaulters from the RNTCP re-treatment regimen (cases), with one consecutively-selected non-defaulter per case. Patients who interrupted anti-tuberculosis treatment for >2 months were classified as defaulters.

**Results:**

1,141 defaulters and 1,189 non-defaulters were included. The median duration of treatment prior to default was 81 days (25%–75% interquartile range 44–117 days) and documented retrieval efforts after treatment interruption were inadequate. Defaulters were more likely to have been male (adjusted odds ratio [aOR] 1.4, 95% confidence interval [CI] 1.2–1.7), have previously defaulted anti-tuberculosis treatment (aOR 1.3 95%CI 1.1–1.6], have previous treatment from non-RNTCP providers (AOR 1.3, 95%CI 1.0–1.6], or have public health facility-based treatment observation (aOR 1.3, 95%CI 1.1–1.6).

**Conclusions:**

Amongst the large number of re-treatment patients in India, default occurs early and often. Improved pre-treatment counseling and community-based treatment provision may reduce default rates. Efforts to retrieve treatment interrupters prior to default require strengthening.

## Introduction

India accounts for nearly 20% of the global incidence of TB, with an estimated annual incidence of 1.9 million tuberculosis (TB) cases [1]. Under the Revised National TB Control Programme (RNTCP), treatment outcomes for new patients, have met or exceeded international targets; over 85% of the 553,660 new smear-positive patients notified in 2006 were successfully treated [2].

The treatment outcome “default” under RNTCP is a patient who has not taken anti-TB drugs for 2 months or more consecutively after starting treatment [3]. The public health and clinical consequences of TB treatment default are severe. Relative to those who complete treatment, patients who default may perpetuate TB transmission and have high post-treatment mortality and rates of recurrent disease [4], [5]. Among new patients, the proportion of patients who defaulted from RNTCP has been relatively low (<8%). Risk factors for default among new patients have been reported in several studies from India [6], [7], [8], [9].

In India, nearly one-quarter of TB patients are notified as “re-treatment”—i.e. with at least one-month history of previous anti-TB treatment [2]. This group is extremely diverse, with patients having been treated with varying durations and anti-TB regimens. These patients may have been treated many years prior or may have only recently failed or defaulted, and may have been treated by the private or public sectors (or both) in the past [10]. Compared to new patients, re-treatment TB patients have much worse treatment outcomes, due to high rates of default. In 2006, among 259,059 notified re-treatment cases, 39,699 (15.3%) were reported to have defaulted [2] when compared to default rates of 7% among new cases. No information has been reported about the profile of these re-treatment defaulters, timing of default, or the extent of programme efforts to retrieve defaulters. Furthermore, risk factors for default may differ from new patients.

We conducted a case-control study to understand the basic clinical and demographic characteristics of patients who defaulted from RNTCP re-treatment regimens, assess the patterns and timing of treatment interruptions, and evaluate risk factors for default.

## Methods

### Treatment of TB in India [3]

In India, patients are evaluated for TB using a standard diagnostic algorithm, starting with sputum-smear microscopy examinations for persons suspected of TB. Patients who are smear-positive are immediately initiated on treatment, and those who are smear-negative are evaluated further with antibiotic trial, repeat sputum examination, radiograph, and clinical evaluation.

All patients diagnosed with TB are evaluated for history of previous anti-TB treatment and those with ≥1 month of treatment from any source are classified as re-treatment cases and prescribed the WHO-recommended Category II treatment, directly-observed and dosed thrice-weekly. RNTCP uses a 3-month (36 doses) intensive phase (IP) of isoniazid, pyrazinamide, rifampicin, and ethambutol (with streptomycin for the first 24 doses), and a 5-month (66 doses) continuation phase (CP) of isoniazid, rifampicin, and ethambutol. If a patient remains smear-positive after IP, then the same is extended by 1 month. Treatment duration is determined by the number of doses taken; missed doses lead to a longer total duration of treatment.

A standard TB treatment card is maintained for every TB patient at the nearest health unit. The card records necessary contact, demographic, clinical, and treatment information, and completed by the treating medical officer.

Adherence to RNTCP treatment is ensured for all TB patients by the use of directly-observed treatment (DOT), wherein an observer watches and supports the patient in taking their drugs. All patients are given a choice to choose their DOT provider who may be either a health worker in the public sector health-care facility or a community volunteer. Community volunteers may include local teachers, private providers, practitioners of traditional medicine, or anyone other than a family member. In the case of re-treatment patients, the need to provide injectable drug during the first 2 months of treatment limits the choice of DOT provider to those practitioners who are legally allowed to administer injectable drugs, i.e. public sector health-care workers, non-public sector health-care workers, or registered medical practitioners.

The RNTCP has well defined guidelines on how to retrieve TB patients who interrupt treatment [11]. Retrieval actions are by home visits and or by phone calls if that information is recorded on the treatment cards. The first retrieval action is to be conducted by the DOT provider, within 24 hours of a missed dose. If that is unsuccessful then it is reported to the next level of supervisors (i.e., multi-purpose paramedical health workers, medical officers), and the local medical officer is responsible for organizing retrieval of the patient within 1 week, followed by the TB programme staff (senior treatment supervisor or TB health visitor) within 2 weeks. Retrieval actions are required to be documented on a dedicated section of the individual patient treatment card.

### Design and Sampling

For this retrospective case-control study, cluster sampling was used to generate a nationally representative sample while retaining operational feasibility of data collection; clusters were the basic TB programme management unit (tuberculosis unit–“TU”) each covering ∼500,000 population. A sample size of 922 cases and 922 controls was determined to be sufficient to achieve 80% power (with a design effect of 2 used to account for single-level cluster sampling), with an odds-ratio >2 for the hypothesized risk factor of missing 5 doses prior to default, estimated to occur among 5% of non-defaulter controls. Given that on average 11 re-treatment patients defaulted per TU per year in 2006, we estimated that 90 TUs would be required to achieve the sample size. All 2,401 TUs were listed; 564 TUs (23.4%) were excluded due to security or operational reasons. From the remaining 1,837 TUs, 90 (4.9%) were randomly selected which were distributed in 73 districts across 15 of the 35 States/Union Territories.

### Definitions and Patient Selection

We defined ‘cases’ as patients registered in 2006 as re-treatment case (treatment category II) with a recorded treatment outcome of default from the TB register. RNTCP defines default as a patient who has not taken anti-TB drugs for more than 2 months consecutively any time after starting treatment [3]. From the selected clusters, all patients who met the case definition were enrolled. Controls were patients registered as a re-treatment case who did not default or die during treatment, and were also selected from the TB register. Patients who died were excluded because of the absence of complete adherence information. For every case of default identified, the next consecutively registered re-treatment patient who did not die or default was selected as a control. The type of re-treatment TB (relapse, failure, treatment after default or re-treatment others] as recorded by the providers at the time of starting treatment on the TB treatment card and TB registers as per RNTCP guidelines was used to classify patients [3].

### Data Source, Collection and Analysis

The data sources used were TB treatment cards and TB registers. Retrospective data collection was limited to information available on routine records, including basic clinical and demographic information, DOT provider type, history of previous treatment, treatment adherence, adverse reactions, and retrieval actions. Treatment adherence was assessed from routine recording of missed doses on treatment cards. Data was abstracted from RNTCP TB registers and TB treatment cards by programme staff into Microsoft Excel, and then analyzed with SPSS 11.0 (SPSS Inc, Chicago, USA) and Epi-Data 2.2. Differences in proportions between defaulters and non-defaulters were compared using the chi-square test (or Fisher's exact test if <10 observations were there in any cell). In multivariate logistic regression, we pre-selected variables for inclusion in the model based on prior information from studies from other settings on risk factors for default from new patients, with consideration for information available on routine records [5], [6], [7], [12], [13], [14], [15], [16], [17], [18]. Information on adverse events was excluded from the multivariate model due to concerns for reporting bias. Information on missed doses was excluded from the multivariate model as that would not be available at the beginning of treatment, hence would be of no value as a predictive factor. Variables were assessed for co-linearity (none was observed), and model fit assessed using the maximum likelihood ratio.

### Ethical Issues

This retrospective analysis was conducted after review and approval by the Central TB Division, Directorate General of Health Services, Ministry of Health and Family Welfare, Government of India. The activity was determined to be programme evaluation of the implementation of national guidelines. Electronic databases created for this analysis were stripped of personal health identifiers and maintained securely.

## Results

### Data Collection

**Figure 1:**
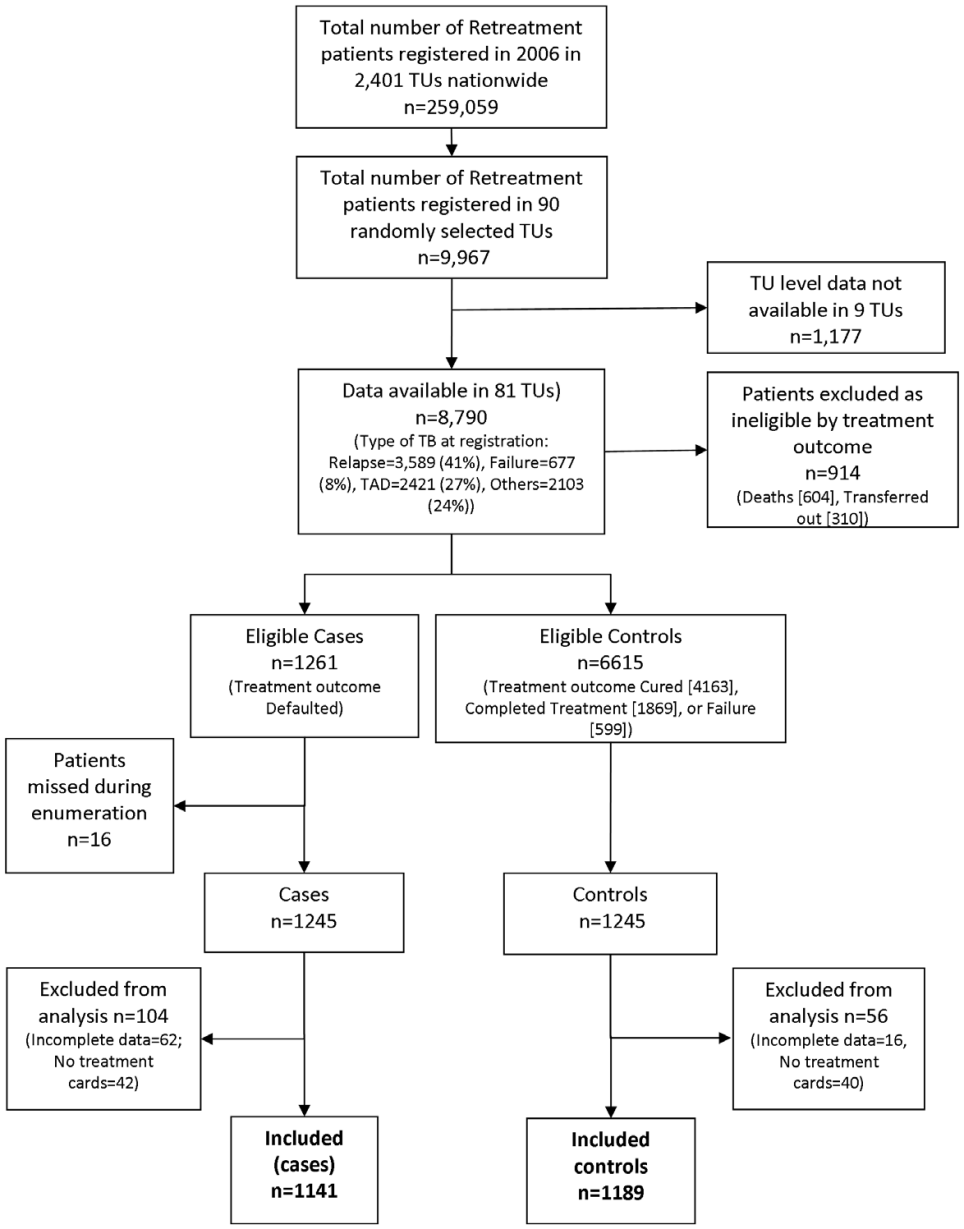
Selection of sampling units and enrollment of cases and controls. Abbreviations: TU = tuberculosis unit (basic programme management unit, used as cluster sampling units), TAD = registration type “treatment after default”.

Out of 90 randomly selected TUs for this study, we received data from 81 (90%), encompassing 2,490 patients **(** **figure 1** **)**. Data could not be collected from 9 TUs due to operational reasons such as loss of treatment cards, TB registers or due to non-availability of staff for data collection. 160 (6.4%) patients were excluded from the analysis due to incomplete patient information (78 patients) or non-availability of treatment cards (82 patients). Of the 2,330 patients included in the final analysis, 1,141 patients were cases and 1,189 patients were controls (including 773 cured, 352 treatment completed and 64 failure cases).

### Characteristics of Patients Who Defaulted

Of the 1,141 patients who defaulted from treatment, 907 (80%) were males (**table 1**). The median age was 38 years (range 7–89 years), and 79.4% were sputum smear-positive. Relatively few patients (102, 8.9%) were registered as re-treatment after failure; in comparison to 429 (38%) classified as re-treatment after default. Nearly one-third (358, 31%) had previous treatment from a source other than RNTCP. Health facilities served as DOT providers for 829 (73%), with the rest served mainly by private practitioners or community DOT providers.

**Table 1:** Demographic and clinical characteristics of re-treatment TB patients, defaulters (cases, N = 1,141) and non-defaulters (controls, N = 1,189), and bivariate risk factors for default, India 2006.

Characteristic	Sub-category	Defaulter n (%)	Non–Defaulter n (%)	Odds Ratio (95% confidence interval)	p-value
Sex	Male	907 (79.5%)	853 (71.7%)	1.56 (1.28–1.89)	<0.01
	Female	234 (20.5%)	336 (28.3%)	Referent	
Age group	<15 years	7 (0.6%)	17 (1.4%)	0.44 (0.17–1.07)	0.05
	15–24 years	131 (11.5%)	166 (14.0%)	0.85 (0.63–1.14)	0.26
	25–34 years	267 (23.4%)	288 (24.2%)	Referent	
	35–44 years	321 (28.1%)	291 (24.5%)	1.19 (0.94–1.51)	0.13
	45–54 years	219 (19.2%)	242 (20.4%)	0.98 (0.76–1.26)	0.84
	55–64 years	150 (13.1%)	116 (9.8%)	1.39 (1.03–1.89)	0.26
	>65 years	46 (4.0%)	69 (5.8%)	0.72 (0.47–1.10)	0.11
Classification	Pulmonary, Smear positive	906(79.4%)	914(76.8%)	Referent	
	Pulmonary, Smear negative	201(17.6%)	221(18.6%)	0.92 (0.74–1.14)	0.42
	Pulmonary, Smear unknown	9(0.7%)	14(1.2%)	0.65 (0.26–1.6)	0.31
	Extra-pulmonary	25 (2.2%)	40 (3.4%)	0.63 (0.37–1.08)	0.07
Type of TB	Relapse	376 (33.0%)	450 (37.8%)	Referent	
	Failure	102 (8.9%)	105 (8.8%)	1.16 (0.85–1.60)	0.33
	Treatment after default	429 (37.6%)	364 (30.6%)	1.41 (1.15–1.72)	<0.01
	Others	234 (20.5%)	270 (22.7%)	1.04 (0.83–1.30)	0.74
Source of previous Treatment	RNTCP	451 (39.5%)	533 (44.8%)	Referent	
	Non-RNTCP	358 (31.4%)	323 (27.2%)	1.31 (1.07–1.60)	<0.01
	Data missing	332 (29.1%)	333 (28.0%)	1.18 (0.96–1.44)	0.10
Nature of DOT provider	Public health facility	829 (72.7%)	786 (66.1%)	Referent	
	Community provider	155 (13.6%)	159 (13.4%)	0.92 (0.72–1.19)	0.52
	Medical college	29 (2.5%)	62 (5.2%)	0.44 (0.28–0.71)	<0.01
	Private provider	90 (7.9%)	130 (10.9%)	0.66 (0.49–0.88)	<0.01
	Non-governmental organization	34 (3.0%)	49 (4.1%)	0.66 (0.41–1.05)	0.06
	Data missing	4 (.4%)	3 (.3%)	1.26 (0.24–7.11)	0.75
Missed doses during intensive phase of treatment*	1 or more	453 (39.7%)	337 (28.3%)	1.66 (1.39–1.99)	<0.01
	2 or more	379 (33.2%)	270 (22.7%)	1.69 (1.40–2.04)	<0.01
	3 or more	322 (28.2%)	224 (18.8%)	1.68 (1.37–2.05)	<0.01
	4 or more	281 (24.6%)	178 (14.9%)	1.86 (1.50–2.30)	<0.01
	5 or more	249 (21.8%)	150 (12.6%)	1.93 (1.54–2.43)	<0.01
	6 or more	216 (18.9%	130 (10.9%)	1.90 (1.49–2.42)	<0.01
	10 or more	126 (11%)	72 (6%)	1.93 (1.41–2.63)	<0.01
Adverse reaction	Documented	119 (10.4%)	5 (0.4%)	27.6 (10.8–76.7)	<0.01
	Not documented	1022 (89.6%)	1184 (99.6%)	Referent	

*Prior to the last dose documented. Sub-categories are mutually exclusive. Referent group for each comparison are those cases and controls with fewer missed doses than the number evaluated.

**Figure 2:**
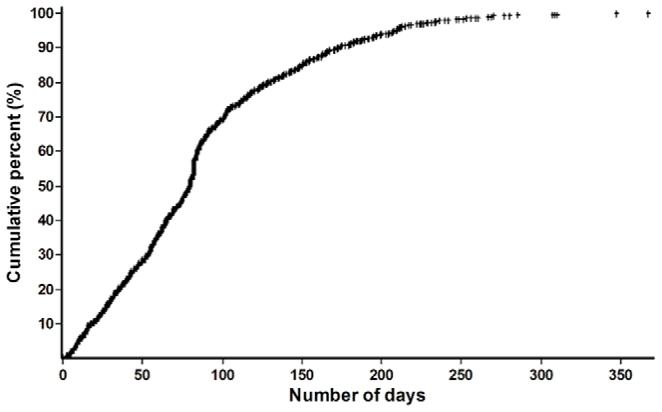
Duration of treatment (in days) completed among re-treatment TB patients who ultimately defaulted from treatment, India 2006 (N = 1,141).

The median duration of treatment prior to default was 81 days (inter-quartile range 44–117 days). Of 1,141 defaulters, 720 (63%) defaulted within 90 days of treatment, prior to completion of 36 doses in IP (**figure 2** **)**; another 281 (25%) patients defaulted after completion of IP but before starting CP, and 140 (12%) patients defaulted during CP.

### Retrieval Actions by the Programme after Treatment Interruptions (Table 2)

**Table 2:** Documented retrieval actions after treatment interruptions among retreatment TB patients who ultimately defaulted from treatment, India 2006 (N = 1,141).

Characteristic	N (%)
Number of retrieval actions	
No retrieval action documented	415 (36%)
At least 1	726 (64%)
At least 2	460 (40%)
At least 3	276 (24%)
At least 4	144 (13%)
Timing of first retrieval action relative to last documented dose (N = 726)	
0–7 days	328 (45%)
8–14 days	87 (12%)
15–21 days	43 (6%)
22–28 days	24 (3%)
>28 days	214 (30%)
Date of retrieval action not mentioned	30 (4%)
Health staff conducting retrieval action (N = 726)	
Staff conducting retrieval action not documented	69 (10%)
TB programme staff	492 (67%)
General health system staff (other than medical officer)	154 (21%)
Medical officer	11 (2%)

Out of 1,141 defaulters, at least 1 retrieval action was documented in 726 (64%) patients. Relative to the last dose taken, the 1^st^ retrieval action was taken within 1 week in only 328 (45%) of defaulters; an additional 87 (12%) patients had retrieval actions during the second week. Out of 726 patients who had any documented retrieval actions, 492 (68%) actions were taken by TB programme staff (senior treatment supervisor or tuberculosis health visitor], and 154 (21%) were done by staff from the general health system. In only 11 (2%) instances was any retrieval action by medical officers (physicians) documented.

### Risk Factors for Default

On bivariate analysis (**table 1**), retreatment patients who defaulted were more likely to be male than female (odds ratio [OR] 1.56, 95% confidence interval [CI] 1.28–1.89). Patients who defaulted were also more likely to have been initially classified as “treatment after default” registration type (OR 1.41, 95% CI 1.15–1.72) or have received previous treatment from a non-RNTCP provider (OR 1.31, 95% CI 1.07–1.60). Patients who defaulted had 28 times the odds (95% CI 10.8–76.7) of having adverse events to anti-TB drugs recorded than patients who did not default.

Treatment interruptions were more common among defaulters. Nearly 40% of patients who defaulted and >28% of the non-defaulters had at least 1 interruption of treatment during IP. Defaulters were more likely to have missed doses, than patients who did not default, regardless of the cut-off chosen for number of missed doses. Five or more missed doses (non-consecutive) or any 3 consecutive missed doses identified defaulters with 79% sensitivity, and 23% specificity.

Multivariate logistic regression (**table 3**) showed that independent risk factors for default included male sex (adjusted odds ratio [AOR] 1.42, 95% CI 1.17–1.73), prior default (AOR 1.22, 95% CI 1.11–1.60), having previous treatment outside RNTCP (AOR 1.28, 95% CI 1.04–1.58), and public health facility based DOT (AOR 1.33, 95% CI 1.11–1.60). Very elderly patients >65 years were slightly less likely to default than our reference age category, (35–44 years).

**Table 3:** Multivariate analysis of risk factors for default among re-treatment tuberculosis patients–India, 2006 (Default cases = 807, Control cases = 853).

Characteristic	Sub-Category	Adjusted Odds Ratio	95% Confidence Limits	P- value
Sex	Male	1.42	1.16–1.73	<0.01
	Female	Referent		
Age	<15 years	0.49	0.19–1.22	0.12
	15–24 years	0.79	0.59–1.04	0.10
	25–34 years	0.87	0.68–1.09	0.23
	45–54 years	0.81	0.63–1.03	0.09
	55–64 years	1.12	0.84–1.51	0.43
	:>65 years	0.60	0.40–0.91	0.02
	35–44 years	Referent		
Type of TB	Failure	1.14	0.84–1.56	0.39
	Treatment after default	1.31	1.07–1.61	<0.01
	Other	0.98	0.77–1.24	0.86
	Relapse	Referent		
DOT Provider	Public health facility	1.33	1.11–1.60	<0.01
	Other facility*	Referent		
Source of prior treatment	Non-RNTCP	1.28	1.04–1.57	<0.01
	Data not reported	1.14	0.92–1.40	0.21
	RNTCP	Referent		

*includes medical colleges, private practitioners, community treatment providers, and non-governmental organizations.

### Reasons for Default Documented

Although the practice was non-systematic, reason for default during the present retreatment regimen was recorded on the TB treatment cards of 735 patients (64.4%). Amongst these 735 patients, the most commonly cited reasons were migration 246 (21.6%), refusal 177 (15.5%), treatment from private sector 101 (8.9%), side effects 110 (9.6%), substance abuse 24 (2.1%), and others (social, HIV, pregnancy) 77 (6.7%).

## Discussion

More re-treatment TB patients are notified in India than any other country in the world, and default among this group is a serious public health problem [19], [20]. This study is the first effort to describe the characteristics and risk factors for default among re-treatment TB patients in India, and the efforts made by RNTCP to retrieve treatment interrupters. In this study, most defaults were seen to occur early, with 88% occurring before starting the CP of anti-TB treatment. The transition between IP and CP appeared to be a particularly vulnerable time for default, with 25% of all defaults occurring in this time period. These findings differ from other settings, which have shown the tendency of default at the later stages of treatment [21].

Independent risk factors for default included male sex, registration as treatment after default, a history of previous anti-TB treatment outside of RNTCP, and DOT at a public health facility. The association of default and male sex has been previously reported for new patients, and it is unsurprising that previous default is associated with default during re-treatment [7], [22]. The association between DOT provision at a public health facility and default has not been reported previously from India. This suggests that limited hours of operation and accessibility—a reality of many health facilities in India—may play a role in non-adherence. RNTCP policies very clearly promote community-based treatment supervision by a DOT provider who is acceptable and accessible to the patient [3]. Although re-treatment patients are treated with streptomycin injections for the first 2 months, local private physicians or registered medical providers—who can legally provide injections—could be better utilized as community-based DOT providers.

While missing doses during IP treatment was modestly associated with default, we found that in practice this would perform poorly as a signal for potential default. For example, nearly 40% of patients who defaulted missed at least one dose during IP, but so did 29% of non-defaulters. Viewed differently, 60% of patients who ultimately defaulted had *no* missed doses in IP documented prior to their default. Hence the usefulness of missing doses in any number or combination as a warning signal for potential default is limited.

The independent risk factors we identified were only weakly associated with default. These findings suggest that profiling registration data to identify and target re-treatment patients at risk for default is not likely to be widely successful. A similar observation on the limited usefulness of patient profiling was made from a case-control study in Hong Kong [17]. Given the high rate of default, efforts to promote treatment adherence should be broadly applied to all re-treatment patients.

This is the first report of RNTCP retrieval efforts for patients who interrupted treatment and ultimately defaulted. Efforts to retrieve treatment interrupters were however found to be weak and far below programme guidelines. Zero retrieval actions were documented in 415 (36%) of the 1,141 defaulters. In those with documented retrieval action in more than half the patients it was delayed by more than 1 week. Contrary to RNTCP guidelines, staff of the general health system minimally participated in default retrieval efforts. For example, although programme guidelines are very clear that default retrieval is chiefly the responsibility of the treating medical officer, ∼2% of the documented retrieval actions were undertaken by medical officers. Instead, 68% of the documented retrieval actions were undertaken by the contractual TB programme staffs, who are few in number and cover large geographical areas. Actions and documentation of retrieval after interruption urgently require strengthening.

### Limitations

By their nature, retrospective analysis based using routine records are subject to different types of information bias. The retrospective nature and the use of existing programme records limited our ability to assess the association of other risk factors which have been previously linked to default among new patients. For example, alcoholism and drug abuse have been previously associated with default in new patients [6], [18]. In this study, however, amongst those patients with a reason for default documented on programme records, substance abuse (including alcohol) was very uncommonly cited. Other risk factors for default in mainly new patients have been described in other settings, such as health knowledge, distance to treatment centre, and patients' economic status; these could not however be assessed in this study [16], [17], [23]. Similarly, we lacked information on events such as prior referral for treatment and migration - both plausible risk factors for default.

Treatment outcomes were not independently validated; if patients who had defaulted were misclassified as completed treatment in programme records, our ability to detect risk factors would have been diluted. The RNTCP, however, maintains a system of ongoing internal programme evaluation and review including periodic validations of recorded data and patient treatment outcomes. Thus widespread misclassification of treatment outcome is unlikely to have occurred. Our finding of a strong association between adverse events and default was likely influenced by reporting bias; hence, this finding should be interpreted with caution. The association between adverse events and default may be overestimated, and we were sufficiently concerned to exclude adverse events *a priori* from our multivariate model.

### Translating Findings into Policy and Practice

RNTCP has begun strengthening several areas to prevent and reduce default in re-treatment patients. RNTCP has enhanced it's monitoring of re-treatment outcomes in programme reviews and internal evaluations. To provide uniform pre-treatment patient education on the need for completing the full course of anti-TB treatment, emphasis during trainings on pre-treatment counseling has been coupled with the development of a standard patient information booklet, distributed in all languages with every box of RNTCP anti-TB drugs. To promote community-based treatment, the Central Unit has begun monitoring the proportion of patients treated by community-based DOT providers. Furthermore, local programme officers are being asked to provide greater emphasis on general health staff involvement with prompt retrieval actions by local providers and monitor treatment provision for patients who migrate during treatment.

### Conclusions

Defaulting from treatment is common among the large number of re-treatment patients in India. Default usually occurs early during treatment, particularly in the transition period from the IP to CP of treatment, and was weakly associated with male sex, prior default, prior treatment from a private provider, and DOT from a public health facility. Efforts to retrieve treatment interrupters fell far short of programme guidelines. Efforts to improve pre-treatment counseling, increase the proportion of patients treated by community-based treatment providers, and strengthen retrieval of treatment interrupters should yield immediate impact on default rates.

This study, however, represents a starting point from where the RNTCP can begin to comprehensively seek to understand and reduce treatment default. Future prospective studies should seek to investigate the roles of other important risk factors that we could not assess in this retrospective analysis. Qualitative research to understand the challenges faced by patients and providers would further aid in pragmatic programmatic decision making.
